# Enhancing Drug Efficacy: A Review of Research Progress in Drug-Loaded Microspheres

**DOI:** 10.7759/cureus.90498

**Published:** 2025-08-19

**Authors:** Yu Zhang, Mohamad Arif Awang Nawi, Ramizu Shaari, Akram Hassan

**Affiliations:** 1 School of Dental Sciences, Universiti Sains Malaysia, Kelantan, MYS

**Keywords:** carrier, chitosan, drug, lipid, microsphere, silica

## Abstract

The efficacy of drugs is intrinsically linked to their performance characteristics. For instance, natural plant extracts, being endogenous chemical constituents of plants, exhibit limited bioavailability and biological activity due to their poor water solubility. When utilized independently, maintaining the continuity of drug action poses a challenge, and the precise targeting of action sites is arduous. This is evident in applications such as inducing osteogenesis at orthopedic surgery sites, providing anti-inflammatory treatment for localized wounds, and enhancing healing processes. With advances in material science, numerous researchers have embarked on the exploration of drug carriers. Techniques include employing microspheres for the prolonged release of drugs, utilizing membrane carriers to fabricate drug-infused dressings that aid in wound healing, and creating solid carriers to facilitate bone defect restoration. This article systematically reviews the varieties, materials, methodologies, outcomes, and advancements in the research of drug-loaded microspheres, offering a foundation for future drug carrier studies. However, it does not offer empirical data support for the selection of clinical drug carriers.

## Introduction and background

Diverse factors, routes, and methods of drug administration, all of which have a direct impact on the degree and rate of drug absorption, can reduce drug efficiency. Most naturally derived plant extracts, such as dandelion sterols, chlorogenic acid, or curcumin, are known to generally have low water solubility, which further affects the activity to which they are applied [1]. For example, the active ingredients of Chinese herbal medicine have been converted into biologically active materials of nanoscale size, characterized by increased water solubility and stability. This advancement should not only save but also increase their biological roles, among which are the functions in promoting osteogenesis and activities against bacteria and antioxidants. Therefore, the selection of the appropriate drug carrier becomes pivotal for drug efficacy. Such drug carriers that perform optimally facilitate controlled drug release, slow metabolism, minimize excretion, prolong drug action, and also amplify therapeutic outcomes. Carriers can be engineered to direct drugs to specific anatomical sites or to selectively target mutated cells, thereby enhancing treatment precision. Additionally, they can improve the stability of the pharmaceutical agent. Following drug release, the polymeric carrier is designed to minimize toxicity and prevent prolonged retention in the body, thereby reducing the risk of accumulation-related adverse effects [2].

The biomaterials showed themselves as excellent carrier materials due to their excellent biocompatibility, non-toxic nature, and easily available sources. Usually, they are divided into two groups: natural and based on the origin of the material. In contrast, the amplified attention in the pharmaceutical field has currently focused on drug carriers, including microspheres and cellular branches, formed with the help of biomaterials [3]. Therefore, this study offers a detailed review of drug delivery microspheres, covering their design rationale, material selection, synthesis methodologies, and the resulting impacts and progress in the field.** **Its purpose is to provide the reference data for future research on carriers and to lay an experimental foundation for clinical carrier selections.

## Review

Chitosan microspheres

Chitosan, a derivative of the natural polysaccharide chitin through partial deacetylation, exhibits a range of physiological properties including biodegradability, biocompatibility, non-toxicity, antibacterial, anticancer, lipid-lowering, and immunity enhancement. Its applications span various domains such as antibacterial agents, medical fibers, dressings, artificial tissue materials, drug sustained-release materials, gene transduction carriers, and tissue engineering, among others, in the biomedical field. Numerous methods exist for synthesizing chitosan microspheres, including spray drying, emulsion cross-linking, coagulation, ionic gelation, high-voltage electrostatic physical cross-linking, and layer-by-layer self-assembly [4]. Depending on the specific requirements, specialized forms like magnetic, adsorbable, and injectable chitosan microspheres can be engineered.

Magnetic Chitosan Microsphere

**Figure 1:**
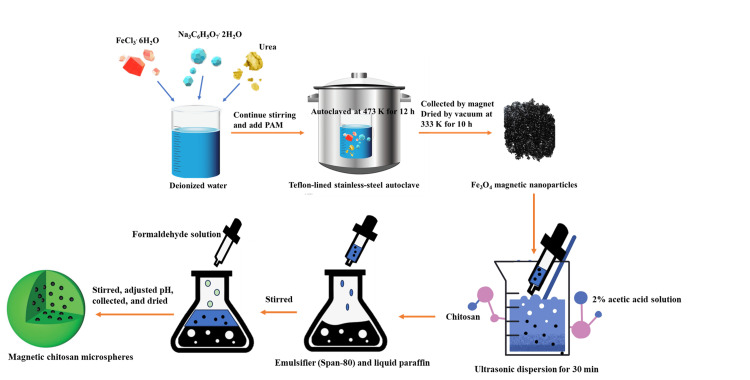
The process of preparing magnetic chitosan microspheres by microemulsion polymerization Image Credit: Authors

Chitosan microspheres are a promising carrier in many biomedical applications since they display mobility orientation in the direction of the magnetic field. A novel form of drug microspheres with magnetic proteins (Figure 1) was prepared in this study. The present investigation designed a microemulsion polymerization process to introduce Fe3O4 magnetic nanoparticles into chitosan microspheres in order to impart pronounced magnetic properties. On examining the beads under the scanning electron microscope (SEM), it was indicated that they were smooth-surfaced microspheres with no defects. The mean diameter was in the order of 2-6 mm, uniformly spherical. These microspheres showed that their surface was smooth, with lots of Fe3O4 magnetic nanoparticles approximately between 200 nm and 300 nm under a transmission electron microscope (TEM) [5]. These were later optimized, and another material, which was pectin, was used for the possibility of an increase in drug absorption for sustained-release formulation. For example, model drugs loaded into magnetic chitosan microspheres were present with an encapsulation efficiency of 85%, loading with metamizole (MtZ). It was carried out in the simulated gastric juice (pH 1.2) and intestinal juice (pH 6.8) under the release process. This, therefore, confirms that the realization of the release test in the F1-F3 formulations to be tested is a pH-dependent process. At pH 6.8, the maximum percentage release of MtZ reached a value of 75% after 12 hours. In addition, the release further reached 91% with an external magnetic field, noting that the phenomenon depends on the magnetic influences [6]. Majeed et al. successfully synthesized the magnetic chitosan-coated halloysite nanotube (mCS-HANT) microspheres for the targeted release of gemcitabine (GC). This showed that halloysite nanotube chitosan microsphere drug carriers could increase the target-to-benefit for the future treatment of breast and bladder cancers [7]. Thus, these findings did prove the particle size to have a major influence on the rate of drug release during the development of chitosan-coated magnetic alginate microspheres (CMAM) using amoxicillin as a model drug. Also, they reported that the encapsulation of the drug in chitosan doubled the release duration of the drug [8]. In another study, chitosan microspheres of magnetic nature (GMS) around 170 µm were developed and cross-linked with glyoxal, followed by the loading and release of the chemotherapeutic drug doxorubicin (DOX) against cancers for prolonged activity by the oil-in-water emulsification technique [9]. The biocompatibility and performance of magnetic chitosan microspheres were confirmed through tests on five different bacterial cultures (*Escherichia coli*, *Staphylococcus aureus*, *Pseudomonas aeruginosa*, *Enterococcus faecalis*, and *Klebsiella pneumoniae*), indicating no inhibitory effect on the bacterial cultures when the concentration of magnetic particles ranged from 10 mg to 30 mg [10].

Adsorbable Chitosan Microsphere

**Figure 2:**
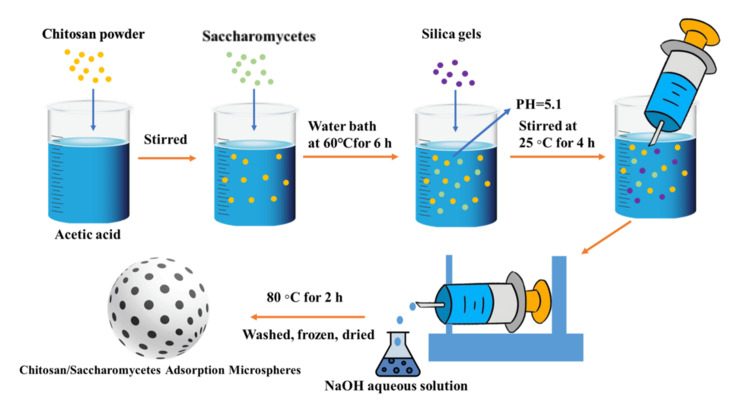
The production process of adsorption chitosan microspheres NaOH: sodium hydroxide Image Credit: Authors

Adsorbable chitosan microspheres have been modified with materials to endow them with a loose and porous structure, enhancing their drug adsorption and release capabilities. Porous chitosan/yeast adsorption microspheres were successfully prepared by Song et al. using silica gel as the pore-forming agent (Figure 2). The optimal mass ratio of chitosan to yeast to silica gel (1:2:3) resulted in the formation of uniformly sized pores (200±10 nm) on the surface and within the microspheres, as revealed by SEM images of the overall, surface, and cross-sectional views, following the dissolution of silica gel in an alkaline medium [11]. Jiang and Hu's modification of chitosan microspheres with rice porous starch (RPS) produced a porous morphology, and it was demonstrated that the adsorption of catechin (CT) by RPS/chitosan involves physical adsorption, chemical adsorption, or strong surface complexation, with the adsorption capacity primarily dependent on the chitosan content, thereby maintaining particle morphology [12]. Furthermore, citric acid cross-linked chitosan microspheres were synthesized using chitosan and citric acid via a microwave-assisted heating method, showing efficient Cr (VI) adsorption from water [13]. Chitosan-alginate microspheres were confirmed to exhibit the highest adsorption capacity for anthocyanins at pH 8.0 [14]. In another study, it was observed that AgCl in the chitosan matrix could be further enhanced, using an emulsion method, to improve the immobilization of AgCl (AgCl@CM) [15]. The as-prepared microspheres were found to be effective for the adsorption of iodide ions (Q). Meanwhile, by the sol-gel phase transformation process, a chitosan/cellulose nanocomposite adsorbent with a porous network structure, entrapping activated carbon, has also been developed, experimentally proving its excellent performance in adsorption [16]. In another approach, thiourea-modified chitosan (TCS) particles are prepared by inverse emulsion dispersion. Among TCS particles, the adsorption of Pt (IV) and Pd (II) was maximum at pH 2.0. TCS showed selective adsorption for these metals in mixtures [17].

Injectable Chitosan Microsphere

**Figure 3:**
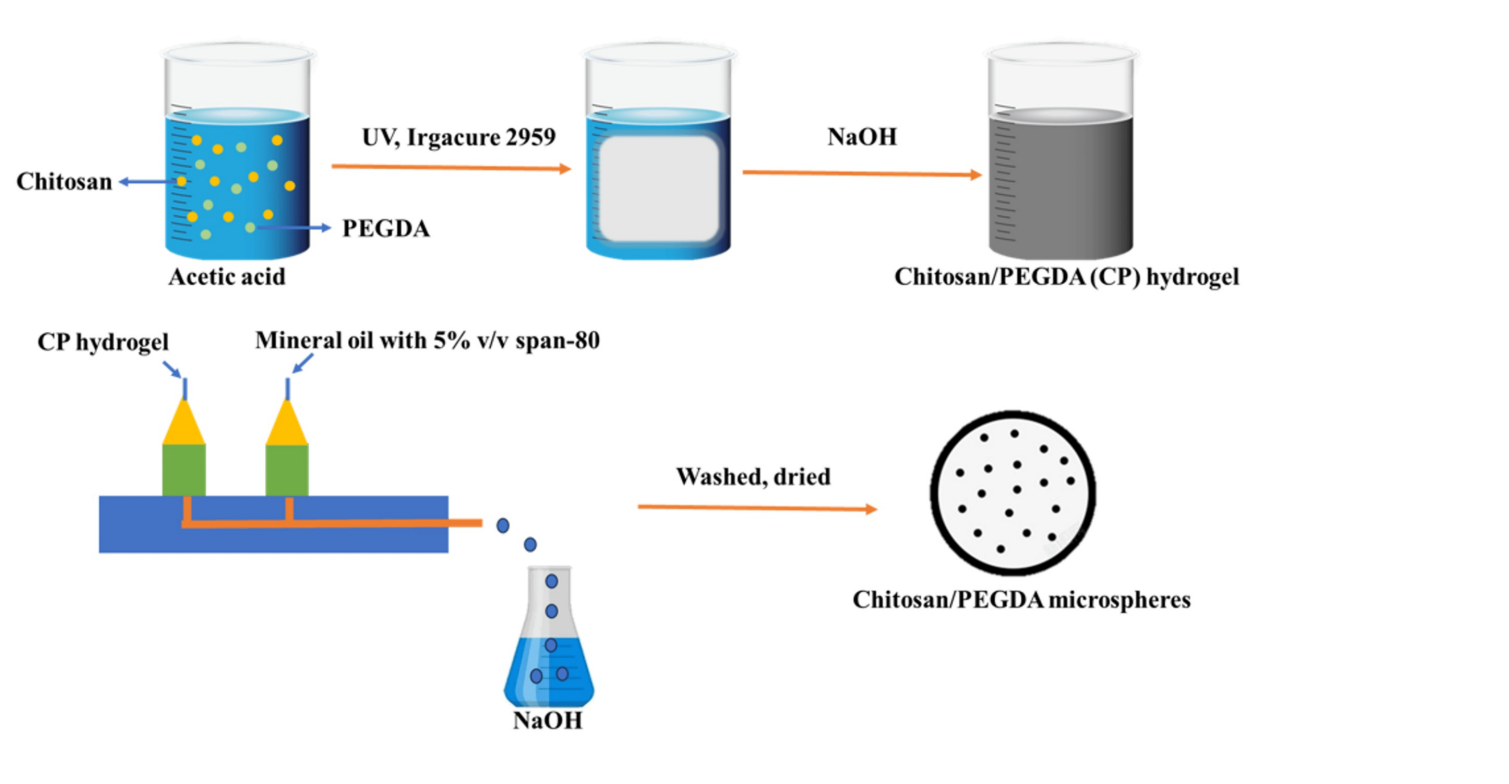
The production process of injectable microspheres UV: ultraviolet; PEGDA: polyethylene glycol diamine; NaOH: sodium hydroxide Image Credit: Authors

Injectable chitosan microspheres are primarily granular micron hydrogel polymers that, at room temperature, exist as an injectable fluid. Upon temperature variation, these microspheres transform into a colloid with limited flowability and adequate structural support, facilitating cell attachment and growth. Moreover, these microspheres enable localized drug delivery. Through microfluidic technology, Lin et al. [18] prepared chitosan/polyethylene glycol diamine (PEGDA) hydrogel microspheres (CP-MS) with a controllable particle size using a water-in-oil method followed by photocross-linking and physical cross-linking (Figure 3). Scanning electron microscopy revealed that the microspheres exhibit a nanoporous surface structure with an average diameter of 397±146 nm, ranging from 300 nm to 500 nm. When chondrocytes were introduced to CP-MS, they demonstrated favorable cell viability and proliferation over extended culture periods. Additionally, Yang et al. developed novel macromolecular/microsphere injectable hydrogels (CMC ODex NPs) integrating carboxymethyl chitosan (CMC) and oxidized dextran nanoparticles (ODex NPs), which exhibit dual functions of drug release and lubrication. This had shown the sustained-release drug behavior of the drug-loaded hydrogels, known as biocompatible hydrogels [19]. Bone morphogenetic proteins (BMPs) are famous for their positive charge, and accordingly, this property of BMP was used in tandem with the negative charge of O-carboxymethyl chitosan microspheres (CMCSM) for controlled release. These microspheres and the gel together form injectable tissue engineering constructs with a dual function of regeneration and anti-infection [20]. In another specific case, several study groups have developed an injectable drug delivery system with the potential to modulate macrophages. In the system, the general concept involves porous chitosan microspheres apart from hydroxypropyl chitin (HPCH) hydrogel. Dimethyloxalylglycine (DMOG) was loaded in the thermosensitive hydrogel of HPCH, and kartogenin (KGN) was conjugated with chitosan microspheres to further be in favor of the proliferation of the local macrophage, boosting cartilage regeneration [21]. Furthermore, chitosan microspheres through an injectable route have been found to possess an antitumor property. Chitosan microspheres have been fabricated by Zhao et al. with the help of electrospinning along with liquid nitrogen. The combination of paclitaxel (PTX) with cis-dichlorodiammine platinum (CDDP) was highly active when given along with 5-fluorouracil (5-FU), demonstrating the significant inhibition of osteosarcoma cell lines HOS and MG-63 [22].

Lipid microspheres

Lipid microspheres are a form of the dispersion system of nanoscale particles and are used for the preparation through dissolving or dispersing drugs in the lipid matrix. Considering the outstanding safety, stability, and biocompatibility of the lipid microspheres, they serve to be an ideal drug delivery carrier. It will be able to bring forth huge reductions in irritations and adverse reactions that come from drugs. Additionally, drugs included in such a particle might be designed to have an extended release. Solid lipid nanoparticles (SLN) were the first-generation lipid microsphere. Subsequent developments in this technology gave rise to the development of nanostructured lipid carriers (NLCs) with markedly enhanced performance. One of the major components in the manufacturing of lipid microspheres includes long-chain triglyceride (LCT) and medium-chain triglyceride (MCT), lecithin, among other additives [23]. LCT and MCT are crucial in providing the necessary fatty acids for the synthesis of lipid microspheres. LCT requires carnitine transport for mitochondrial metabolism, and an overdose can lead to accumulation in the liver, lungs, and other body parts after long-term use [24]. Conversely, MCT engages directly in mitochondrial metabolism without the need for carnitine, which may alleviate certain liver functions in patients with acute liver dysfunction [25]. Thus, the selection of raw materials for lipid microsphere synthesis must be customized according to the specific condition of the patient. While there are various methods for producing lipid microspheres, high-pressure homogenization (HPH) is identified as the most common technique [26]. Additional methods include solvent emulsification-evaporation, melt emulsification, and solvent emulsification-diffusion, providing a range of options for the synthesis process [23].

**Figure 4:**
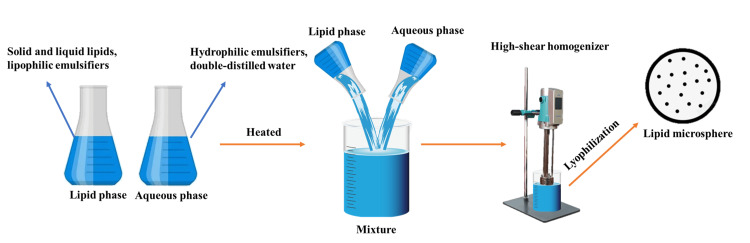
The process of producing lipid microspheres using high-pressure homogenization Image Credit: Authors

To facilitate subsequent research and the selection of suitable lipid materials, this article provides a summary of the materials and applications of lipid microspheres, as reported in the reference literature over the past five years (Table 1). A research team prepared docetaxel (DT) and curcumin co-loaded nanostructured lipid carriers (DTCR NLCs) using an HPH technology (Figure 4). The lipid phase comprised glyceryl palmitostearate, trimyristin, MCT (22:63:15), phospholipon 90GVR, PEG 4000 monostearate, and stearylamine. The hot lipid phase was added to a hot surfactant aqueous solution of Solutol HS 15 VR to prepare the pre-lotion, and DTCR NLCs were produced using a high-pressure homogenizer. In vitro experiments have confirmed that DTCR NLCs demonstrate good stability and drug release performance, enhancing the efficacy of taxanes in non-small cell lung cancer treatment [27]. Further, Gu et al. developed phloretin-loaded NLCs using the HPH method. The lipid phase was comprised of 6% decaglyceride monoester and 6% Tween 80 co-surfactant, along with phloretin, and the aqueous phase. The average particle size of the prepared NLC of phloretin was 137.40±3.27 nm. The in vitro release study of phloretin NLC revealed that the medium received a sustained-release amount of loaded phloretin [28]. Another group has prepared NLCs using other lipids, cetyl palmitate and Gelucire®, this time carrying DOX and subjected to ultrasonic heat treatment. The lipid phase contained solid lipid, oleic acid, polysorbate 80, and DOX. Comparative study between the two showed that NLC formulated with cetyl palmitate as a solid matrix gave significantly bigger results compared to those formulated with Gelucire®. Both the NLC formulations exhibited significant inhibitory activity against the proliferation of breast cancer cells [29]. Llorente et al. utilized active essential oils as liquid lipids to prepare riluzole (RLZ) NLC using the HPH method. A lipid phase containing RLZ, solid lipid (beeswax), and liquid lipids (a 50% blend of lavender and mint oil) was prepared, along with an aqueous phase containing added surfactants (Tween® 80). The resulting RLZ NLC was demonstrated to continuously release RLZ, suggesting its potential for treating hyperproliferative skin diseases [30]. Finally, Alam et al. prepared NLCs of isradipine for myocardial infarction treatment, selecting solid lipid Emulcire 61 (containing magnetite) and liquid lipid Capryol 90 for the lipid phases, while the aqueous phase was prepared by dissolving 3% of surfactants Tween® 80 and Poloxamer 188 in a 2:1 ratio in distilled water [31]. Curcumin was encapsulated in NLC by other scholars for the localized treatment of skin diseases. A lipid phase consisting of Precirol® ATO 5, Labrafac® lipophile WL 1349, Tween®, and curcumin was prepared, along with an aqueous phase containing Poloxamer 407 dissolved in ultrapure water [32]. Li et al. developed NLCs loaded with docosahexaenoic acid (DHA) aimed at treating peri-implantitis in rats. The lipid phase was comprised of monostearic acid, squalene, DHA, and two surfactants (Tween 80 and glycol), with the aqueous phase being ultrapure water [33]. In ophthalmology, Mo et al. prepared an NLC gel to enhance the ocular delivery of dexamethasone. The lipid phase included soy lecithin, dexamethasone, soybean oil, and glycerin, while the aqueous phase was formed by dissolving Pluronic F68 in deionized water. Experimental results have indicated that the NLC significantly enhances the corneal permeability of dexamethasone and prolongs its retention time on the corneal surface [34]. In oncology, Qiu et al. demonstrated the effectiveness of RNA therapy using lipid nanoparticles (LNP) in the preclinical models of lymphangioleiomyomatosis (LAM). Lipidoid nanoparticles were created by rapidly mixing active lipidoids, cholesterol, 1,2-dioleoyl-sn-glycero-3-phosphocholine (DOPC) or 1,2-dioleoyl-sn-glycero3-phosphoethanolamine (DOPE) or 1,2-distearoyl-snglycero-3-phosphocholine (DSPC), and mPEG2000-DMG in ethanol solution with a sodium acetate buffer containing mRNA, utilizing the NanoAssemblr microfluidic system [35].

**Table 1:** Raw materials and applications for the production of lipid microspheres DOPC: 1,2-dioleoyl-sn-glycero-3-phosphocholine; DOPE: 1,2-dioleoyl-sn-glycero3-phosphoethanolamine; DSPC: 1,2-distearoyl-snglycero-3-phosphocholine

Lipid microsphere	Lipid phase	Aqueous phase	Drug	Application	References
Docetaxel and curcumin co-loaded nanostructured lipid carriers	Glyceryl palmitostearate, trimyristin, medium-chain triglyceride, phospholipon 90GVR, PEG 4000 monostearate, and stearylamine	Solutol HS 15 VR hot surfactant aqueous solution	Docetaxel, curcumin	Non-small cell lung carcinoma	Rawal et al., 2020 [27]
Phloretin nanostructured lipid carriers	Decaglyceride monoester, Tween 80, co-surfactant, phloretin	Deionized water	Phloretin	Antioxidant, anti-inflammatory, anticancer	Gu et al., 2022 [28]
Doxorubicin nanostructured lipid carriers	Cetyl palmitate or Gelucire®, oleic acid, polysorbate 80, doxorubicin	HCl buffer	Doxorubicin	Breast cancer	Moraes et al., 2021 [29]
Riluzole nanostructured lipid carriers	Riluzole, solid lipid (beeswax), liquid lipids (mixture of 50% lavender and 50% mint oil)	Deionized water (Tween® 80)	Riluzole	Proliferative skin disease	Llorente et al., 2023 [30]
Nanostructured lipid carriers of isradipine	Solid lipid, Emulcire 61 (containing magnetic bead), liquid lipid, Capryol 90	Distilled water (Tween® 80 and Poloxamer 188)	Isradipine	Myocardial infarction	Alam et al., 2022 [31]
Curcumin nanostructured lipid carriers	Precirol® ATO 5, Labrafac® lipophile WL 1349, Tween®, curcumin	Ultrapure water (Poloxamer 407)	Curcumin	Skin disease	Calderon‐Jacinto et al., 2022 [32]
Docosahexaenoic acid nanostructured lipid carriers	Monostearic acid, squalene, docosahexaenoic acid, two surfactants (Tween 80 and glycerol)	Ultrapure water	Docosahexaenoic	Peri-implantitis	Li et al., 2023 [33]
Dexamethasone nanostructured lipid carriers	Soy lecithin, dexamethasone, soybean oil	Deionized water (glycerol and Pluronic F68)	Dexamethasone	Ophthalmology	Mo et al., 2018 [34]
Lipid nanoparticles loaded with mRNA	Active lipidoids, cholesterol, DOPC or DOPE or DSPC, and mPEG2000-DMG	Sodium acetate buffer containing mRNA	mRNA	Lymphangioleiomyomatosis	Qiu et al., 2022 [35]

Silica microspheres

**Figure 5:**
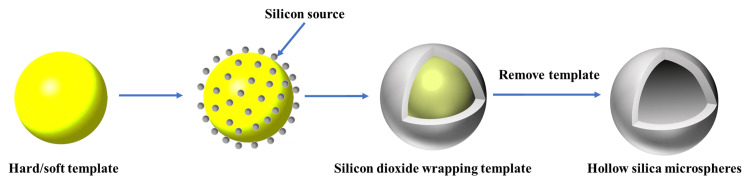
The process for producing hollow silica microspheres using the template method Image Credit: Authors

Silicon dioxide, a high-performance inorganic material, has been extensively studied in the field of nanomaterials due to its biocompatibility, stability, and ease of modification, finding applications in drug release, capsule encapsulation, nanocatalysts, and more. Currently, hollow silica microspheres are widely utilized. The preparation methods for these microspheres include the template method, spray drying method, and microfluidic method [36]. However, the spray drying and microfluidic methods are often avoided due to their complexity, their slowness, or the production of highly polydisperse products. The template method (Figure 5) is now the most prevalent for creating hollow silica microspheres, classified into hard and soft template methods based on the template material and its characteristics [37]. The hard template method employs a rigid template for silica coating, followed by template removal through calcination or solvent treatment to form a shell structure. Polymer latex particles, particularly polystyrene (PS), are favored in the hard template method for producing hollow silica [38]. Other porous resin beads (Amberlite XAD7HP) have been used as a rigid template for the preparation of nickel catalysts supported on silica microspheres by the alternate adsorption of nickel nitrate and tetraethoxysilane (TEOS) precursors on the template [39]. Li et al. designed the work of synthesizing the MoS2 nanosheet microsphere and forming an aluminum shell with porous silicon on the surface. Thioacetamide and sodium molybdate dihydrate (Na₂MoO₄·2H₂O) were uniformly dispersed in deionized water under continuous stirring. The polyethylene solution was further mixed with propylene glycol F68 under vigorous stirring. The solution obtained was then taken in a Teflon-lined stainless steel autoclave and held at 180°C for a time period of 12 hours. The powder obtained was washed and dried to afford MoS2 microspheres acting as a template for the fabrication of MoS2@SiO2 microspheres, showing the enhanced electrocatalytic activity and stability of this material during experiments [40]. For instance, other researchers used resin nanospheres as hard templates for the fabrication of hollow silica microspheres, in which yolk@shell hollow nanoparticles with an AuPt alloy were further synthesized by the fast aerosol process. These nanoparticles are referred to as AuPt@SiO2, and they are favorable for the catalytic activity in the epoxidation of styrene [41]. The soft template method, recognized for its simplicity in template removal, is a prevalent approach for creating hollow nanomaterials. Currently, the most frequently employed soft templates encompass microemulsion templates, micellar vesicle templates, and bubble templates, all existing in fluid forms, allowing for the template's direct removal prior to the reaction's conclusion. Zhao et al. employed a lotion droplet template interface comprising liquid paraffin and tetraethyl orthosilicate (TEOS) to initiate a hydrolytic condensation reaction. Subsequent removal of the paraffin with ethanol resulted in the formation of hollow silica microspheres. Their investigations revealed that the ethanol concentration in the water phase was a critical determinant of the microspheres' average diameter, with the silica wall thickness also varying with the emulsifier content, hydrophobic chain length, TEOS concentration, and catalyst activity [42]. Tian et al. introduced a novel method to synthesize bioinspired nano-core-shell silica microspheres (CSSM) using tetrapropyl orthosilicate as the silicon source and phenolic resin as the soft template. The microspheres were found to have diameters ranging from 150 nm to 340 nm and shell thicknesses between 25 nm and 83 nm, exhibiting notable hydrophilicity [43]. Furthermore, a team utilized 3-aminophenol/formaldehyde (AF) resin as a soft template, TEOS as the silicon source, and hexadecyltrimethylammonium bromide (CTAB) to fabricate silica particles, which demonstrated effective adsorption of Cr (VI) [44].

Silica microspheres find numerous applications in the medical domain. A group of researchers prepared silica microspheres loaded with nitrofurazone (NFZ) and lidocaine (LD), designed via the sol-gel method of Box-Behnken, in line with this recommendation. Tests revealed that the release rates of NFZ and LD were 30% and 33%, respectively, after eight hours. The microspheres achieved drug release while significantly reducing drug crystallinity. In wound healing applications, Sivakumar et al. confirmed that a mixed scaffold containing ferulic acid-loaded silica microspheres exhibited effective antibacterial activity against common wound pathogens [45]. Regarding drug delivery, Gao et al. developed hollow mesoporous silica microspheres loaded with curcumin (HMSMs@curcumin) to enhance curcumin's oral bioavailability [46]. Wang et al. synthesized a novel composite scaffold of mesoporous silica microspheres, nano-hydroxyapatite, and polyurethane loaded with levofloxacin, demonstrating its potential in treating chronic osteomyelitis in rabbits [47]. Furthermore, Zhou et al. prepared photosynthesized silica microspheres (PSM) and radiolabeled them with Lutetium-177 (177Lu), showing that 177Lu PSM possesses excellent in vivo radiolabeled stability and antitumor activity [48].

Other types of microspheres

Polymers, including polypropylene (PP), are widely utilized in microsphere fabrication [49] due to their chemical resistance, heat resistance, electrical insulation, high-strength mechanical properties, and excellent wear resistance. Few researchers have used PP in the synthesis of microspheres for various applications [50]. In addition, microspheres made of poly(DL-lactic-co-glycolic acid) (PLGA) and poly(propylene fumarate) (PPF) have been developed for the controlled delivery of bioactive molecules. Scaffolds developed from these microspheres demonstrated sustained release for at least up to 28 days [51]. In addition, PLGA microspheres containing BMP-2, combined with 3D scaffolds, have been reported to possess the potential to release growth factors slowly for an enhanced effect of bone regeneration [52]. The very low rate of biodegradation of polymers in the body remains a critical area of research due to its significant impact on clinical applications [53]. Inorganic microspheres, such as titanium dioxide, glass, calcium carbonate, ferric oxide, and hydroxyapatite, are also notable in this field [54]. The biodegradation rate of these polymers in the body is very low, and currently, this is a critical field under study with respect to clinical applications. In addition, Qiu et al. further synthesized a series of porous calcium carbonate microspheres (CCMS) with bovine serum albumin (BSA) as an additive and loaded camptothecin through diffusion and adsorption. These CCMS loaded with camptothecin showed pH-dependent release and continuous inhibitory activity on cancer cell growth in vitro [55]. Organic microspheres such as theophylline-loaded starch, albumin, and gelatin microspheres exhibited diverse applications compared to chitosan and lipid microspheres. Sodium trimetaphosphate (STMP) was utilized as a cross-linking agent to prepare anionic cross-linked starch microspheres, with methylene blue as a model drug, demonstrating that these starch microspheres could sustain drug release. In another study, tramadol hydrochloride was encapsulated in gelatin microspheres, achieving a high encapsulation efficiency of 97.2%, which facilitated controlled and prolonged drug delivery [56].

## Conclusions

All types of microspheres have distinct properties, but those used as drug carriers should exhibit effective sustained-release properties. Literature reviews indicate that microspheres, particularly hollow ones, have achieved substantial success in drug release performance due to their enhanced capabilities in drug adsorption and encapsulation. This paper extensively covers hollow silica microspheres, emphasizing the need to consider the availability, economic feasibility, and biocompatibility of materials used in microsphere fabrication. Importantly, for in vivo drug administration, the biodegradability of the microsphere materials, especially natural organic materials like chitosan and lipids, is crucial due to their favorable biodegradability. This paper provides a detailed summary of the production methods, materials, and research advancements in chitosan and lipid microsphere synthesis, aiming to guide future research. Enhancing the degradability of inorganic and polymer materials is a key research focus. Additionally, ensuring the stability of drug delivery microspheres is paramount for sustained drug release. Therefore, future microsphere preparation should prioritize material selection, biodegradability enhancement, and microsphere stability.
